# IMplementing best practice post-partum contraceptive services through a quality imPROVEment initiative for and with immigrant women in Sweden (IMPROVE it): a protocol for a cluster randomised control trial with a process evaluation

**DOI:** 10.1186/s12889-023-15776-8

**Published:** 2023-05-03

**Authors:** Helena Kilander, Rachael Sorcher, Sofia Berglundh, Kerstin Petersson, Anna Wängborg, Kristina Gemzell- Danielsson, Karin Emtell Iwarsson, Gunnar Brandén, Johan Thor, Elin C. Larsson

**Affiliations:** 1 Department of Women’s and Children’s Health, Karolinska Institutet, and the WHO Collaborating Centre, Karolinska University Hospital, Stockholm, Sweden; 2 Jönköping Academy for Improvement of Health and Welfare, School of Health and Welfare, Jönköping University, Jönköping, Sweden; 3 Department of Global Public Health, Karolinska Institutet, Solna, Sweden; 4 Department of Clinical Sciences, Obstetrics and Gynaecology, Umeå University, Umeå, Sweden; 5 Center for Epidemiology and Social Medicine, Region Stockholm, Sweden

**Keywords:** Cluster randomized control trial, Co-design, Immigrants, Joint learning, Post-partum contraception, Process evaluation, Quality Improvement Collaborative

## Abstract

**Background:**

Immigrant women’s challenges in realizing sexual and reproductive health and rights (SRHR) are exacerbated by the lack of knowledge regarding how to tailor post-partum contraceptive services to their needs. Therefore, the overall aim of the IMPROVE-it project is to promote equity in SRHR through improvement of contraceptive services with and for immigrant women, and, thus, to strengthen women’s possibility to choose and initiate effective contraceptive methods post-partum.

**Methods:**

This Quality Improvement Collaborative (QIC) on contraceptive services and use will combine a cluster randomized controlled trial (cRCT) with a process evaluation. The cRCT will be conducted at 28 maternal health clinics (MHCs) in Sweden, that are the clusters and unit of randomization, and include women attending regular post-partum visits within 16 weeks post birth. Utilizing the Breakthrough Series Collaborative model, the study’s intervention strategies include learning sessions, action periods, and workshops informed by joint learning, co-design, and evidence-based practices. The primary outcome, women’s choice of an effective contraceptive method within 16 weeks after giving birth, will be measured using the Swedish Pregnancy Register (SPR). Secondary outcomes regarding women’s experiences of contraceptive counselling, use and satisfaction of chosen contraceptive method will be evaluated using questionnaires completed by participating women at enrolment, 6 and 12 months post enrolment. The outcomes including readiness, motivation, competence and confidence will be measured through project documentation and questionnaires. The project’s primary outcome involving women’s choice of contraceptive method will be estimated by using a logistic regression analysis. A multivariate analysis will be performed to control for age, sociodemographic characteristics, and reproductive history. The process evaluation will be conducted using recordings from learning sessions, questionnaires aimed at participating midwives, intervention checklists and project documents.

**Discussion:**

The intervention’s co-design activities will meaningfully include immigrants in implementation research and allow midwives to have a direct, immediate impact on improving patient care. This study will also provide evidence as to what extent, how and why the QIC was effective in post-partum contraceptive services.

**Trial registration:**

NCT05521646, August 30, 2022.

## Introduction

Globally, as compared to native-born women, immigrant women across countries of all income groups report lower access to contraceptive services, accompanied by higher rates of unintended pregnancies and abortions [1–4]. A plethora of barriers to accessing contraceptive services exist for immigrants, such as language challenges, various cultural and religious beliefs, strict gender roles, social pressure and financial factors [5, 6]. Additionally, despite variation across immigrant populations, their family planning knowledge is generally low, causing misconceptions about contraceptive methods and limiting their possibilities to prevent unintended pregnancies [7–9].

Immigrants’ contraceptive decision making is further influenced by migration status, changes or instabilities in living conditions, and adaptation to resettlement communities [10–12]. While immigrants post resettlement might employ using contraceptives to regain financial and social stability, barriers to contraceptive services still exist [12–15].

With this said, there is a paucity of research regarding immigrants and post-partum contraception use. Ensuring access to effective post-partum contraception is fundamental when seeking to reduce inequities that immigrants face in SRHR, as it prevents unintended pregnancies, abortions and enables birthspacing [16]. A short interpregnancy interval (IPI) – less than 12 months – increases the risk of complications, e.g., preterm birth, low birthweight, stillbirth and neonatal death [17, 18]. Use of more effective post-partum contraception, such as long-acting reversible contraception (LARC) (i.e. intrauterine devices) and short-acting reversible contraception (SARC) (i.e. oral contraceptive pills), supports birth-spacing and prevents unintended pregnancies, abortions as well as pregnancy-related adverse events [19]. What little evidence exists regarding immigrants’ post-partum contraceptive use highlights that immigrant women are less likely to plan or attend post-partum clinic visits and use reliable contraception methods in a timely manner compared to non-immigrants, sometimes despite their interest [20–22].

The antenatal and post-partum period offers room for strengthening SRHR by improving contraceptive services. One focus of such improvement efforts can include double sessions of contraceptive counseling (CC), whereby sessions occur during both the antenatal and the post-partum period, as this has shown to increase contraceptive use after giving birth [23–25].

Additionally, offering information about contraceptive methods’ effectiveness and potential side effects can counteract patient-provider power imbalances that may hinder women’s choices of contraceptive methods [26]. For example, in Sweden, structured CC (“the LOWE model” using an educational video, an effectiveness chart, four key questions, and a box with contraceptive models) increased LARC choice, initiation and use, when controlled for migration background [27]. Immigrant women in Sweden have also expressed the importance of trust in CC encounters, especially since they perceive these as private matters [28].

High-quality post-partum CC—meaning that the HCP discusses what matters most to a patient in choosing a method—occurs less often among immigrant women, who are less likely to know where to seek such services, even if they are free of charge [29, 30]. Furthermore, the needs of immigrant women may be known by HCPs, but the womens’ knowledge and values may not be properly addressed [31]. HCPs express several challenges in communicating with immigrant women regarding SRHR including navigating cultural and religious differences and patients’ distrust in healthcare [31–33]. Overall, previous studies report several opportunities for improving underutilized SRHR services by interacting with immigrant women, taking into account their experiences, and adapting one’s practice to the situation at hand [34–36].

Several quality improvement collaboratives (QICs) have been applied to improve obstetric, gynaecological and neonatal care, although many lacked close user engagement [37–41]. There is also promising evidence of how the umbrella of community-based participatory research (CBPR) and co-design methods are being employed to address health disparities and improve SRHR services for immigrants [42–44]. Co-design in healthcare involves HCPs, patients, caregivers, designers and researchers working together as equal partners to improve specified health outcomes and system efficiency [45]. Therefore, QICs based on the Breakthrough Series Collaborative model and co-design methods can help organizations to make and sustain measurable improvement according to patients needs, as well as stimulate joint learning and networking among HCPs [46].

In Sweden, a few small-scale QICs have demonstrated the importance and effectiveness of increasing user engagement. The evaluations of these QICs report positive results when seeking to improve access to or choice of effective methods in the context of abortion or post-partum care [40, 41, 47]. The lack of studies similar to these reflect how little evidence exists on how to combine QICs and co-design methods to improve immigrants’ post-partum contraceptive services.

**Fig. 1:**
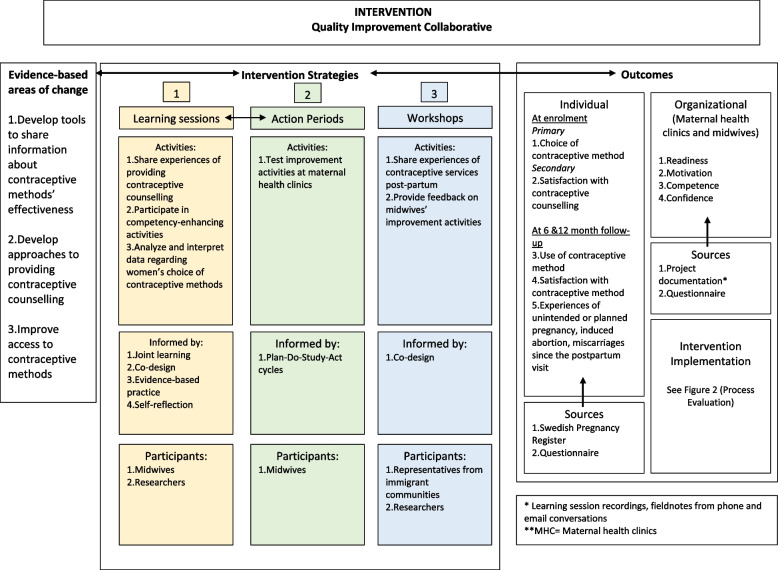
Intervention. Quality improvement collaborative

Therefore, the overall aim of IMPROVE-it is to promote equity in SRHR through the improvement of contraceptive services and, thus, to strengthen women’s possibility to choose and initiate effective contraceptive methods post-partum. The hypothesis is that focusing on the following three evidence-based areas of change regarding contraceptive services:

- 1) developing tools to share information about contraceptive methods’ effectiveness,
- 2) developing approaches to providing CC and
- 3) improving access to contraceptive methods (Fig. 1) will lead to a significant increase in immigrant women’s choice and use of effective contraceptive methods.

### Research questions

How does a Quality Improvement Collaborative (QIC) combined with co-design methodology influence immigrant women’s choice of effective post-partum contraception?

How and by what mechanisms does a QIC influence contraceptive services, and how does context affect the QIC efforts and outcomes?

## Methods

### Study design

The IMPROVE-it project will combine a cluster randomized controlled trial (cRCT) with a process evaluation to develop and study the in-depth effects of an intervention, a QIC, on contraceptive services and use. Harnessing QICs and co-design methods supported by National Quality Registers can ultimately help to close gaps between ordinary clinical practice and evidence-based guidelines [46, 48–51].

Figure 1shows the program theory of the project, which is based on a pilot study [47]. The project’s primary outcome is women´s choice of an effective contraceptive method within 16 weeks after giving birth. Effective contraception is defined as LARC (i.e. intrauterine devices and subdermal implants) and SARC (i.e. oral contraceptive pills, rings, patches, and injections).

### Study setting

In 2021, 30% of newborn children in Sweden had a mother who was born outside of Sweden [52]. The foreign-born population in Sweden is heterogenous, with some of the most common countries of birth for persons born outside of Europe being: Syria, Somalia, Iraq and Afghanistan [53]. In this study, refugees and asylum seekers are considered immigrants (foreign-born persons). Post-partum clinic visit(s), including contraceptive services available to all residents as part of public health services, should be offered within 16 weeks post-partum [54]. Midwives are the main providers of post-partum care and also administer the majority of contraceptive methods [55]. This cRCT will be conducted at 28 MHCs in the regions of Jonkoping, Vastra Gotaland and Stockholm. In 2021, these 28 MHCs had between 1900–3300 pregnant women yearly [56]. All MHCs in Jonkoping (22 MHCs, 3812 births) and Stockholm (94 MCHs, 26 191 births) were invited to participate. In Vastra Gotaland (94 MCHs, 17 595 births), we invited selected clinics of sufficient cluster size.

### Strategies used for recruitment

Managers at MHCs were contacted by co-authors EL or HK during 2019–2020 to inquire about their willingness to take part in the IMPROVE-it study. Due to the Covid-19 pandemic, the project’s commencement was postponed.

Initially, 29 MHCs accepted the invitation, finally 28 MHCs in three regions decided to participate. Informational meetings were held between the research team and the midwives at the MHCs during spring and fall 2021 before randomization was conducted.

### Eligibility of MHCs and inclusion criteria

Inclusion criteria: MHCs in the previously described regions with > 100 registered pregnant women during 2021 were invited to participate in the cRCT.

Intervention clinics commit to register women’s choice of contraception in the Swedish Pregnancy Register (SPR) during the post-partum MHC visit and participate in the QIC for 12 months [56]. The control clinics commit to provide routine care and register consenting women’s choice of contraception in the SPR.

### Self-administered questionnaire

Inclusion criteria: Women 18 years and above coming for post-partum visits within 16 weeks post birth (live birth).

Exclusion criteria: Women who do not anticipate becoming sexually active with a male partner within six months.

Midwives at intervention and control clinics will invite women to participate in the study, i.e. respond to a digital or printed paper questionnaire during the visit and be contacted for follow-up six and 12 months post enrolment. There is also the possibility to participate in a telephone interview with an interpreter. Additional information will be provided at the MHCs before prospective participants give their written, digital or verbal informed consent.

### Data management

A safe web application (REDCap) for builing and managing questionnaires will be used to secure data storage [57]. The web application allows the women who wish to participate to register personal data such as name, social security number, address, email address and mobile number via a QR code. These personal data will be pseudonymised, and the research subjects will receive a study ID. Paper questionnaires will be manually entered into the REDCap database. Three of the researchers (SB, KEI and AW) will promote data quality (e.g., prevent double data entry and conduct range checks for data values).

### The intervention – a QIC initiative

Midwives at the intervention clinics were invited to participate in the QIC initiative and gave their written consent to participate in the entire project. Midwives who joined the QIC after the first learning session (LS) were informed before participating in an LS. Through the consent process, participants agreed for LSs to be digitally recorded.

The intervention strategies presented at LSs will be applied at participating MHCs over nine months in 2022–2023 and follow those of the Breakthrough Series Collaborative model including [47]:

- Four remote digitally run and recorded learning sessions (LS) every third month.
- Action periods between every LS where participants apply learnings and evaluate changes in their MCH.
- Co-design workshops with members of the target population.

The LSs will be informed by joint learning, self-reflection, co-design and evidence-based practice. At each LS, midwives in the intervention group will participate in competency-enhancing activities, exchange experiences about counselling as well as analyze and interpret data from the SPR regarding women’s choice of contraceptive methods. They will also decide on improvement activities that they would like to test in clinical practice during the action periods by using Plan Do Study Act (PDSA) cycles [58, 59]. Through the LSs, HCPs will also practice self-reflection, which has been described as an important factor in a previous study when seeking to facilitate co-design of health and related services [60]. Such sessions will also contribute to building trusting relationships between providers of various regions, a context factor found crucial to the implementation of successful continuing midwifery care [61].

Throughout the intervention, representatives from immigrant communities will be invited to co-design workshops with researchers to share their experiences of contraceptive services post-partum and provide feedback on midwives’ improvement activities, such as the development of visual aids for counselling. Personas will also be developed during these workshops to enable midwives to best understand and design services to meet the diverse needs of their patients [62].

### Defining outcomes and data collection methods

Reporting of the IMPROVE-it study results will follow the Standard Protocol Items for Clinical Trials (SPIRIT) [63, 64].

#### cRCT

The SPR will be used to retrieve the primary outcome, women’s choice of contraception, and covariates for evaluating social determinants such as age, sociodemographic characteristics and reproductive history. To evaluate secondary outcomes regarding participants’ satisfaction with the contraceptive counselling received, women attending the post-partum visit will complete a questionnaire that utilizes the Person-Centered Contraceptive Counseling Measure-scale (PCCC-scale) [65]. Participating women will also receive a follow-up questionnaire six and 12 months after the postpartum visit to evaluate their ongoing contraceptive use and satisfaction with the contraceptive method. In addition, the follow-up questionnaire will ask about unintended or planned pregnancy, induced abortion and miscarriages since the post-partum visit (Table 1).

**Table 1:**
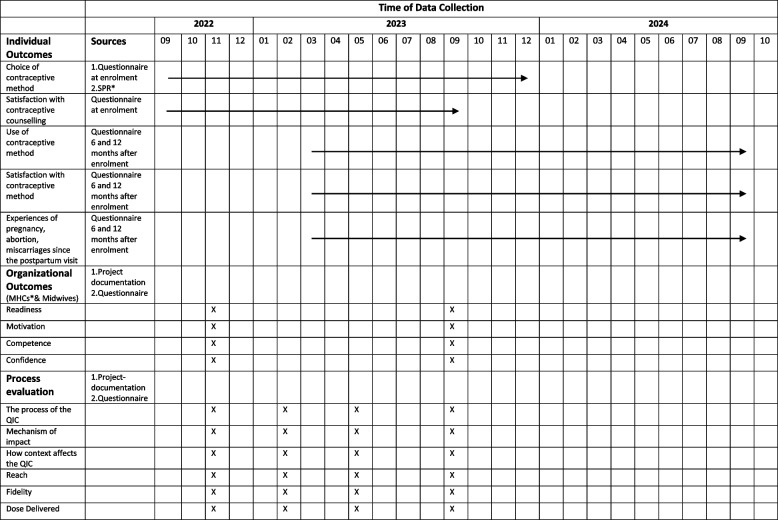
Time of data collection during the randomised control trial and process evaluation

^*^SPR Swedish Pregnancy Register

^*^MHCs Maternal health clinics

The questionnaire will be completed digitally, manually on paper, or via telephone by interpreter amongst women who are illiterate or prefer this method. The printed paper questionnaire has been translated into the nine most common languages spoken by immigrants in Sweden. Participants will chose to receive follow-up text messages, e-mails or paper questionnaires at six and 12 month after the post-partum visit. Those who answered via telephone will be recalled. Several reminders/recalls will be performed.

The organizational outcomes including the MHCs’ and midwives’ readiness, motivation, competence and confidence will be measured through project documentation and a questionnaire (Table 1).

### Power analysis and randomisation

The intraclass correlation coefficient (ICC) was set to 0.05 in the power analysis, equivalent to an observed ICC in a similar study [66]. To detect an anticipated 15 percentage point increase in choosing an effective contraceptive method (SARC/LARC) with 90% power at an α = 0.05 (i.e. the probability of rejecting a true null hypothesis), we needed to include at least 30 MHCs, each contributing an average of 100 participants (pregnant women). However, 28 (out of 52) agreed to participate in the study. This implies a statistical power of about 88% which was considered sufficient. The sample size calculations were performed using Stata version 17.0 [67]. No power calculations were performed for secondary outcomes.

The randomisation procedure followed a blocked cluster design. The MHCs (clusters) were first assigned to groups (blocks) based on their regional affiliation and size (number of visits by immigrant mothers in the past year), resulting in two blocks for Jonkoping and Stockholm and three blocks for Vastra Gotaland. Intervention and control status was then randomly assigned to clusters within each block.

### Statistical analysis

The analysis population will consist of all women in the cRCT. Our primary goal is to estimate the effect of the QIC on choosing an effective contraceptive method using logistic regression analysis, relying on the randomization scheme to retrieve a causal estimate. We will also investigate whether the intervention effect varies by sociodemographic variables and reproductive history.

### Process evaluation

**Fig. 2:**
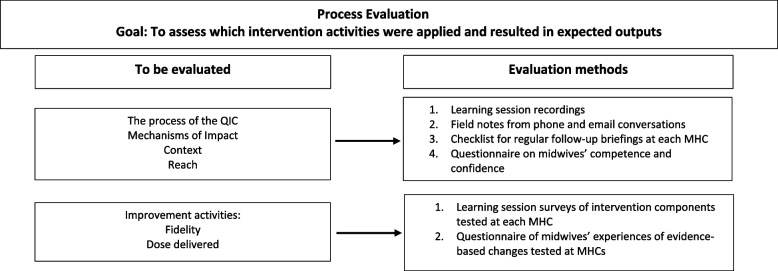
Process evaluation. Goal: to assess which intervention activities were applied and resulted in expected outputs

The process evaluation started during the planning phase of the cRCT. Chosen improvement activities tested during action periods within three evidence-based areas of change, as well as the process itself, will be evaluated (Fig. 2). The study will use both quantitative and qualitative research methods to evaluate the QIC initiative in-depth and aims to discover which actions were carried out, what worked where, and how and why (or why not) an impact occurred.

According to Moore et al. the process evaluation will be divided into three areas for which data will be collected [68]:

- 1. The process of the QIC––to describe what is being applied, we will study and document the preparation phase of the QIC initiative, as well as ongoing activities throughout the cRCT, emerging changes in implementation and experiences of the intervention.
- 2. Mechanisms of impact––to examine how the delivered QIC produced change at the intervention clinics, we will document the evidence-based changes tested, and evaluate their impact on women’s choices, as well as identify what components work and how, including the immigrant women’s contribution in coproducing the contraceptive services.
- 3. Context–– to examine how the context affects the QIC’s implementation and outcomes, we will document how this varies across contexts, and how the intervention is delivered in the various county councils and MHCs. We will also evaluate social processes and different organizational circumstances that enable QICs as well as the target-population’s involvement.

### Quantitative data collection and analysis

To assess fidelity (quality) and dose of codesigned contraceptive services delivered (quantity), a survey will be used at each LS (every third month) that collects data on components of the intervention which have been tested at each MHC. Midwives will also be asked to complete a questionnaire regarding their experiences of evidence-based changes tested at the MHCs. To assess mechanisms of impact, the participating midwives will be asked to fill in a questionnaire on their competence and confidence in providing contraceptive counselling before and after the intervention. Additional data might also be collected as the project will use an emerging design (Table 1).

### Qualitative data collection

During each phase of the QIC, project documents will be collected including recordings from the LSs, field notes from phone meetings and e-mail conversations. Additionally, a checklist will be used for regular follow-up briefings with coordinators at each MHC. These tools aim to capture contextual factors (such as staff rotatation, work load, motivation, management, etc.) facilitating or hindering the QIC intervention at the various MHCs as well as potential unintended outcomes (Table 1).

### Analysis

Quantitative data will be reported as medians and frequencies for the evidence-based changes made at MHCs. Qualitative thematic analysis will be employed to the qualitative data[69].

## Discussion

Many health promotion and prevention studies have been carried out to improve SRHR services. Immigrants have collaborated on several health-related studies but have rarely been involved in all stages of the research process, especially the later implementation phases [42, 70]. Therefore, IMPROVE-it is designed to engage immigrants in the research process, especially in the intervention implementation phase, where representatives from immigrant communities will provide feedback on suggested improvement activities during co-design workshops.

The project will also bring forward women’s voices in that it will invite them to participate in a self-administered questionnaire regarding their choice of contraceptive method and satisfaction during CC at the post-partum visit. Importantly, to accommodate immigrant women’s views especially, we have translated the questionnaires into nine languages. Moreover, women who are illiterate will have the opportunity to participate and report their experiences via telephone with an interpreter. Data from the registers and potentially the self-administered questionnaire will be fed back to the midwives during the LSs as part of the QIC intervention.

Furthermore, the intervention includes co-design activities, including LSs created together with representatives from the target population that will use personas developed from focus group discussion data. This will enable HCPs to self-reflect and engage with immigrant women’s lived experiences of post-partum contraceptive services. Other co-design activities, such as workshops, will enable representatives from immigrant communities to provide feedback on suggested improvement activities regarding three evidence-based areas of change. To better understand the process of how the QIC is perceived, IMPROVE-it will evaluate organizational outcomes such as midwives’ confidence and competence. Ultimately, this intervention allows HCPs to have a direct impact on improving patient care by applying immediate changes to their practice that may be sustained over time.

However, a key limitation of the study is that it may be difficult to capture exactly how the intervention is delivered in the various health services regions and MHCs, since we have to rely on midwives self-reports of tested changes. To overcome this difficulty, we will triangulate our data by also collecting such information via coordinators and LS questionnaires.

Previous case studies reporting on QICs show positive results when seeking to improve access to or choice of effective methods in the context of abortion or post-partum care [40, 41, 47]. Yet, there is still limited evidence regarding to what extent, how and why a QIC is effective and what factors influence a QIC in these healthcare contexts, due to a general lack of understanding on the role of context, mechanisms of change and critical components of success [71–73]. This lack of understanding is especially problematic given the lack of SRHR intervention studies including immigrant women, as we know that their contraceptive use is generally low [1–4].

A challenge of IMPROVE-it will be to evaluate which components have had which effect, but the process evaluation will enable an in-depth analysis of these issues. By using the design of a cRCT, the study also offers the potential to distinguish between varying influences of overlapping improvement activities.

## Data Availability

The dataset generated and/or analysed during the current study is not publicly available due to restrictions from the ethics review board, but it can be made available to qualified researchers upon request, after approval from the ethics board. EL should be contacted to request the data.
